# Visualisation Approaches in Qualitative Metasynthesis for Health Research

**DOI:** 10.7759/cureus.101027

**Published:** 2026-01-07

**Authors:** Aviraj K.S., Sumana Mukhopadhyay, Divya Darshani Sharma, Anmol Goyal, Manvi Sagar, Jobinse Jose

**Affiliations:** 1 Department of Community Medicine, ESIC Medical College and Hospital, Noida, IND; 2 Department of Community Medicine, Grant Government Medical College, Mumbai, IND; 3 Department of Community Medicine, Gian Sagar Medical College and Hospital, Dehradun, IND; 4 Department of Community Medicine, RIMT Medical College and Hospital, Mandi Gobindgarh, IND; 5 Department of Community Medicine, Maharishi Markandeshwar College of Medical Sciences and Research (MMCMSR), Ambala, IND; 6 Department of Community Medicine, Jubilee Mission Medical College and Research Institute, Thrissur, IND

**Keywords:** chord diagram, data visualization, evidence synthesis, gis mapping, network mapping, qualitative meta-synthesis, sankey diagram

## Abstract

Qualitative meta-synthesis, the interpretive counterpart of systematic review, integrates findings from multiple qualitative studies to generate deeper insights into complex health phenomena. Visualisation techniques enhance the interpretability of such syntheses by transforming textual data into clear, relational, and actionable forms. This review showcases advanced data visualisation tools to explore sociocultural influences on pregnancy and childbirth practices across global contexts. Following Preferred Reporting Items for Systematic Reviews and Meta-Analyses (PRISMA) guidelines and a registered PROSPERO protocol, qualitative studies examining cultural beliefs and maternity practices were systematically reviewed and thematically synthesised. The synthesised data were visualised using Sankey, Chord and Network diagrams, as well as geo-spatial sentiment maps, to illustrate thematic transitions, interconnections, and regional variations in sociocultural practices and perceptions. The Sankey diagram depicted the continuity and transition of cultural practices from pregnancy to childbirth. In contrast, the Chord diagram revealed interwoven relationships among themes such as rituals, dietary customs, and support systems across ethnic groups. Network analysis identified central, recurring sociocultural themes like gender roles, emotional support, and traditional beliefs. Geo-spatial sentiment mapping highlighted regional differences, with strong positive sentiments in parts of Africa, moderate neutrality in Asia and Europe, and negative perceptions in South America. Collectively, these visualisations uncovered cultural patterns and regional diversity, emphasising the importance of culturally sensitive, context-specific approaches to maternal health. Visual analytics enhances qualitative meta-synthesis by revealing hidden cultural patterns, improving interpretation, and guiding culturally sensitive health interventions.

## Introduction and background

Within the evidence pyramid, systematic reviews are highly valued. Its qualitative counterpart, Metasynthesis, is a form of evidence synthesis that systematically brings together findings from qualitative research to create deeper and more comprehensive understandings of complex phenomena [1]. It is a rigorous, interpretive methodology that has evolved in the past decades in response to the growing need for synthesising qualitative studies for evidence-based decision-making [2].

Qualitative synthesis follows a structured process that includes defining a research question, systematically identifying and appraising relevant studies. It synthesises findings across multiple sources to generate interpretations that extend beyond individual studies, making the whole greater than the sum of its parts. [3,4]. It is used to explore complex human behaviours and their health impacts, linking common themes to inform the development of theoretical frameworks [5].

Metasynthesis is increasingly valued for its ability to deepen understanding of community perspectives, subjective health experiences, and contextual determinants influencing outcomes. It supports theory building, identifies research gaps, and informs context-sensitive practice and policy, making determinants relevant for clinical, administrative, and public health audiences [6,7]. Methodologically, metasynthesis has evolved from early adaptations of meta-analysis to transparent, protocol-driven approaches that prioritise thematic interpretation, conceptual modelling, generating in-depth hypotheses and the visual representation of relationships across themes and contexts [5-9]. Unlike quantitative meta-analysis, metasynthesis addresses how and why phenomena occur within specific contexts, offering experiential insights that cannot be captured through numerical aggregation alone [10].

Background

**Figure 1:**
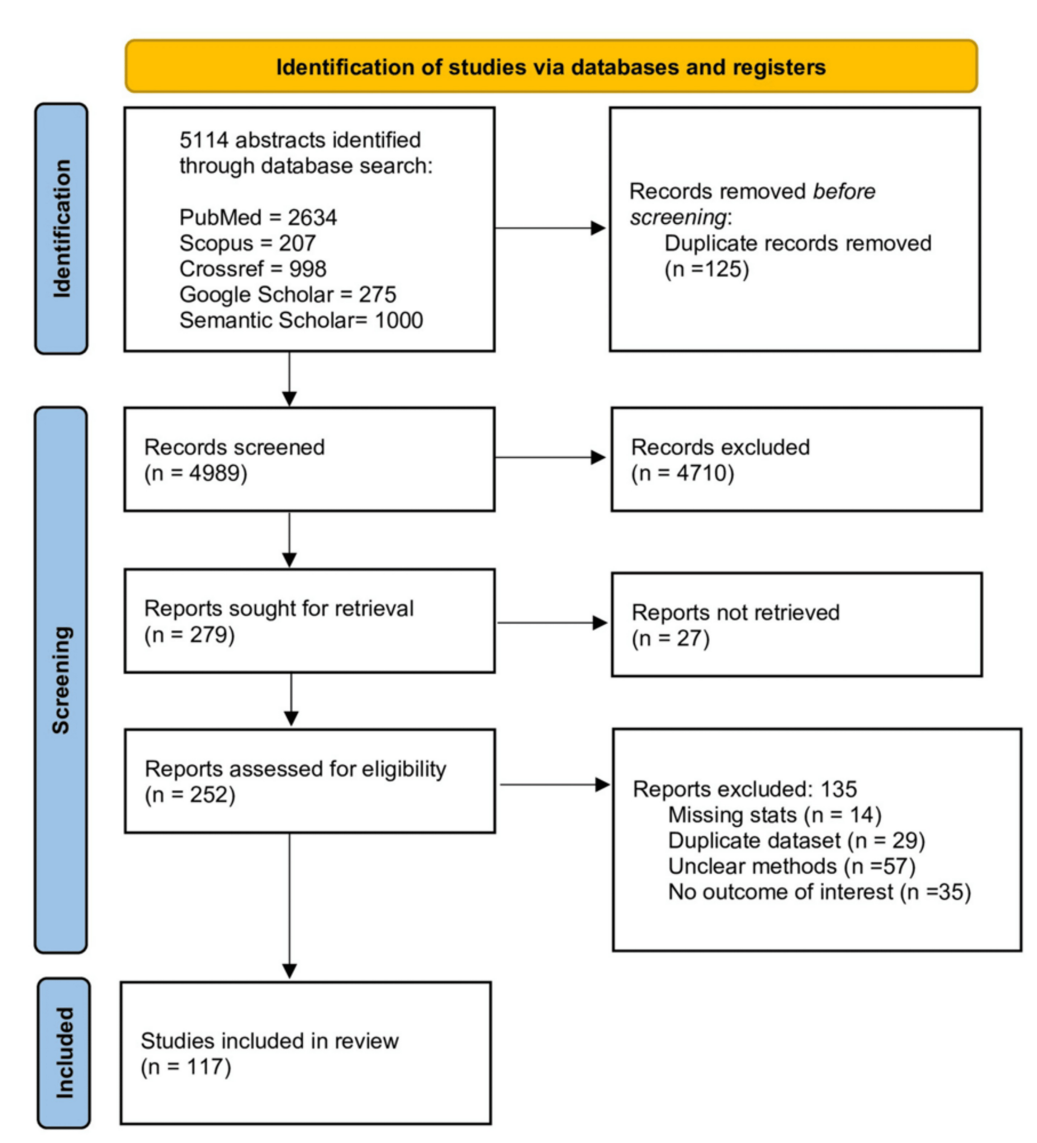
PRISMA flowchart for studies screened and included (n = 117) in the systematic review and metasynthesis. PRISMA, Preferred Reporting Items for Systematic Reviews and Meta-Analyses

We conducted a review adopting a structured interpretative meta-synthesis methodology grounded in Sandelowski and Barroso’s principles to capture socio-cultural influence on pregnancy and childbirth globally. Following PRISMA 2020 and a registered PROSPERO protocol (ID: CRD420251047986), an exhaustive search across multiple databases and grey literature identified qualitative studies exploring cultural beliefs and maternity practices. One hundred and seventeen eligible studies underwent blinded screening in Rayyan, followed by systematic data extraction and inductive coding in NVivo (Figure 1). Coding was conducted independently by multiple reviewers, with discrepancies discussed and resolved through consensus to ensure consistency and analytic rigour. Reflexive discussions were held throughout the synthesis process to acknowledge and minimise interpretive bias. Overarching themes and subthemes were developed using constant comparison, reciprocal translation, and thematic clustering. A key strength of the methodology was the use of advanced visualisation techniques in R. These visualisations transformed complex cultural dynamics into clear, accessible structures, enhancing the interpretability of findings. Various R packages (Ver 4.3.3) made the process seamless once the data extraction and thematic analysis were done in a systematic way as per the Cochrane guidelines. With the use of this research question, we aimed to use various visualisation techniques that complemented the narrative synthesis, making complex cultural dynamics more interpretable for global health audiences.

## Review

Overview of visualisation methods

Sankey Diagram

Originally introduced by Captain Sankey in 1898 to visualise energy flows, Sankey diagrams remain underexplored in healthcare research literature. The diagram is particularly powerful in representing transitions and flows of outcomes within a population or dataset, making it highly suitable for the synthesis and interpretation of complex findings. Three broad categories of use for Sankey were found in the medical literature to (1) visualise flow/transitions over time, (2) depict flow/transitions to specific events, and (3) demonstrate associations [11].

A Sankey diagram is composed of two main components:

Nodes: Typically represented as vertical lines, these indicate the categories or stages in a system.

Arcs (or flows): Shown as broad horizontal bands, these represent the connections between nodes. The width of each arc corresponds to the magnitude of the flow.

In the context of a thematic synthesis in healthcare research, a Sankey diagram can map how individual codes or subthemes (source nodes) link to overarching themes (target nodes). The flow value represents the number of studies in which a particular code or theme was identified.

The Sankey diagram emphasises directionality of thematic influence by establishing a directional flow, displaying prominence. Accordingly, the size of the node and the width of the connecting flow both reflect the relative magnitude or prevalence of the findings. A Sankey diagram can be drawn for discrete variables with interrelated values in any dataset by converting them into the appropriate nodes and arcs format. 

**Figure 2:**
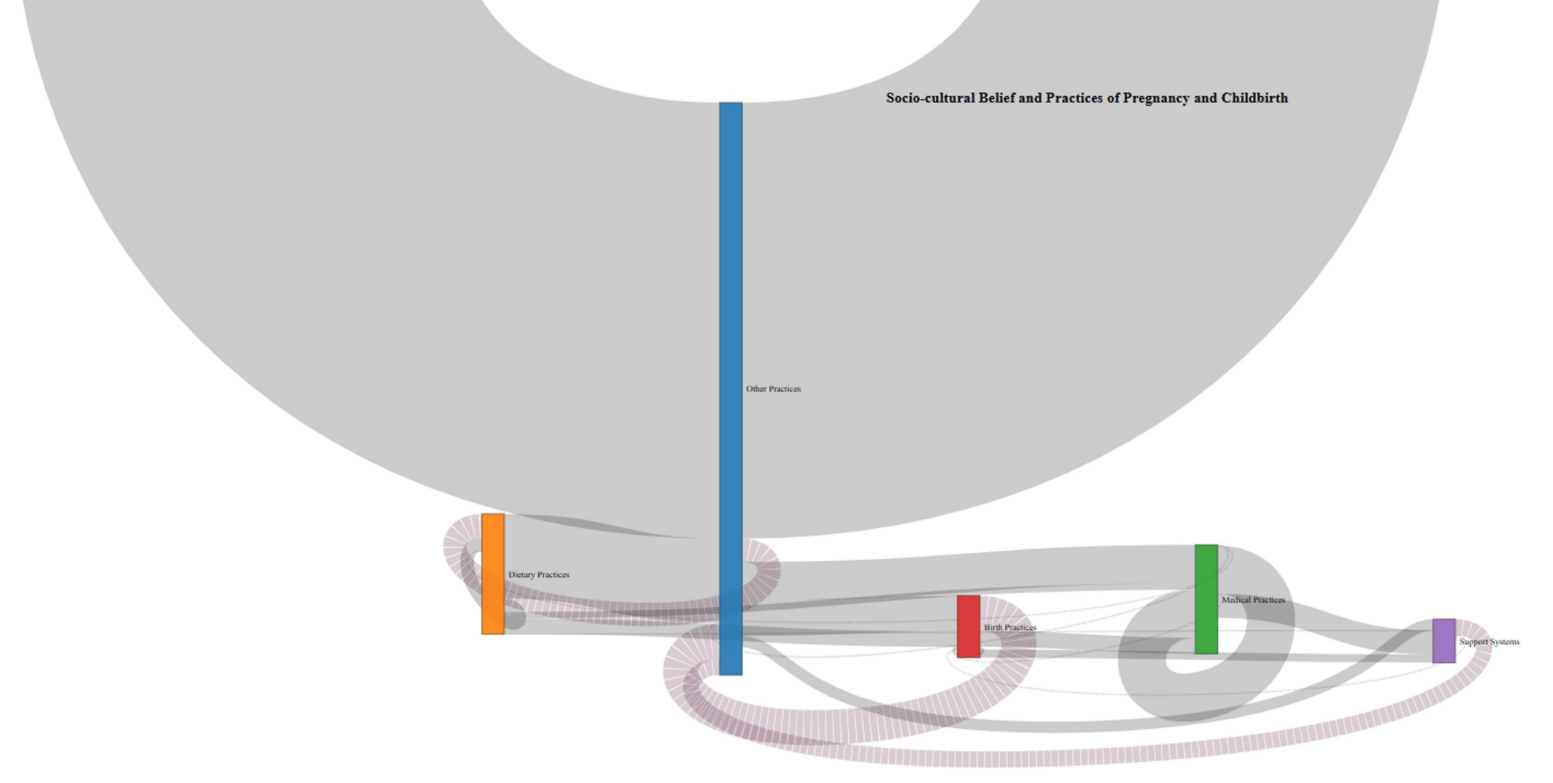
Sankey diagram depicting thematic interpretation of sociocultural beliefs and practices in pregnancy and childbirth.

We utilised a comprehensive qualitative dataset on sociocultural beliefs and practices surrounding pregnancy and childbirth from our systematic review and metasynthesis. Data were systematically processed by importing into R, selecting relevant information, cleaning, aggregating similar practices into categories (e.g., dietary, support systems, medical, birth and other practices), and creating transition data to visualise how practices move from pregnancy to childbirth. A Sankey diagram was then generated, with nodes representing practice categories and links illustrating the frequency of transitions, providing a clear representation of cultural practices across the two phases (Figure 2).

The Sankey diagram highlights several key patterns in sociocultural practices across pregnancy and childbirth. A clear continuity is seen, with dietary practices and support systems persisting strongly across both phases, reflecting deep-rooted cultural adherence. At the same time, a shift is evident, with medical practices gaining greater prominence during childbirth due to the need for immediate healthcare interventions. Importantly, the diagram illustrates the integration of traditional practices with modern medical care, suggesting an adaptive blending that ensures both cultural acceptability and clinical safety. The multiple interconnections among practice categories emphasise the complexity and dynamic nature of maternal healthcare, where cultural beliefs, rituals, and medical advice often intersect. Together, these findings demonstrate that maternal health practices are not static but evolve across phases, balancing tradition with modernity in ways that are critical for culturally sensitive and effective healthcare delivery. Thus, Sankey diagrams provide an intuitive and visually engaging way to track the trajectory of evidence synthesis, making them valuable tools for summarising complex qualitative or mixed-methods research.

Chord Diagram

A chord diagram visualises how different categories are connected. Each category is shown as a section along the circle, and the links or flows between them are shown as curved lines (chords) inside the circle. Chord diagrams have evolved into powerful tools for depicting complex information across disciplines [12]. In healthcare, they are used to depict transitions such as treatment switching or discontinuation, patient referral patterns, or disease associations. In epidemiology, genetics, and large-scale studies, chord diagrams are particularly valuable for mapping disease-disease associations or tracking patient movement across hospitals. Beyond quantitative data, chord diagrams are increasingly applied in qualitative research, where they reveal interconnections among themes, topics, or codes across datasets, and innovations such as document theme mapping have extended their utility to meta-syntheses and bibliometric analyses [13].

**Figure 3:**
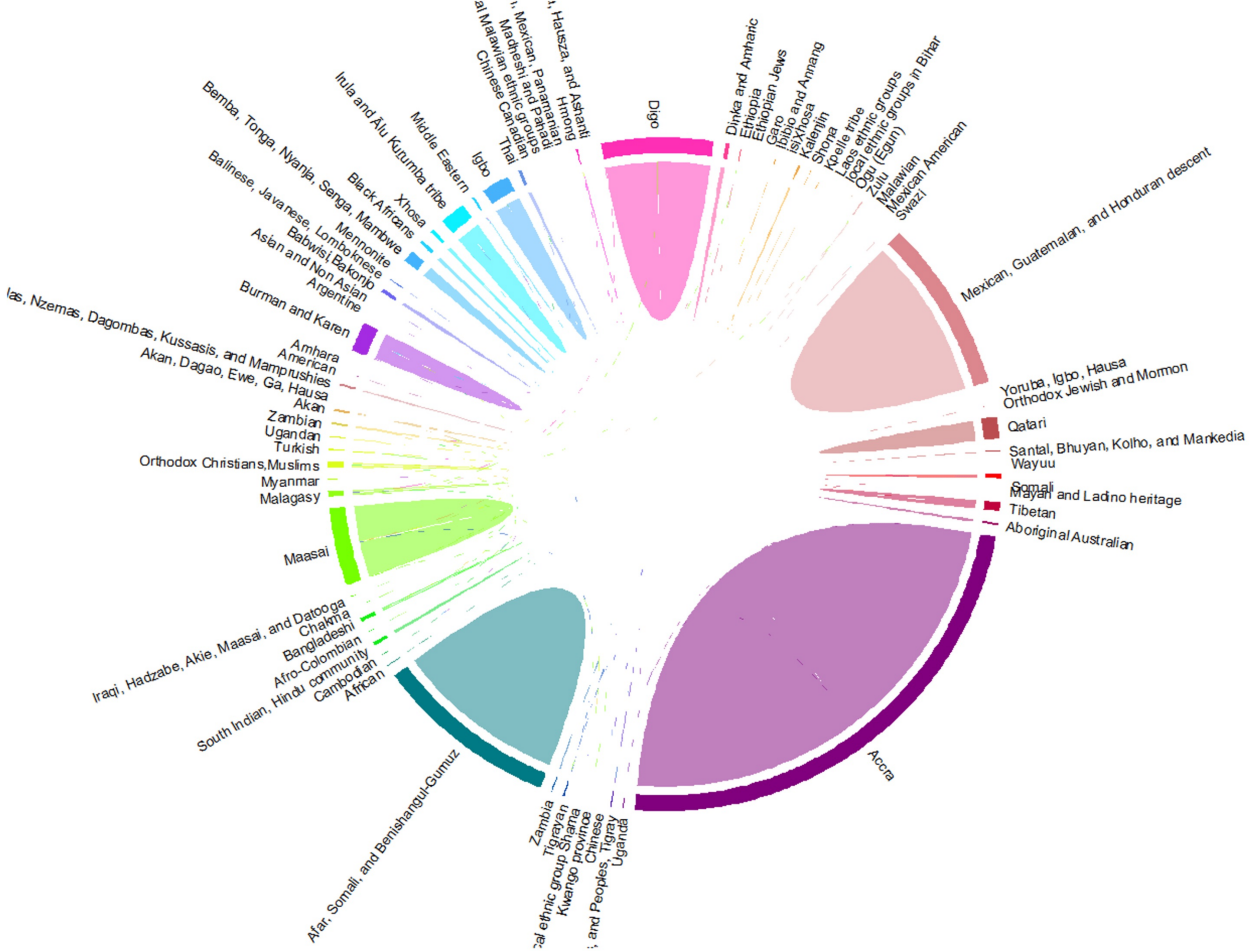
Chord diagram showing distribution of sociocultural themes across geographic locations.

We synthesised the qualitative dataset on sociocultural beliefs and practices related to pregnancy and childbirth, drawn from diverse geographical and cultural contexts. Data were imported into R, where key columns on ethnic backgrounds and themes/subthemes were selected and combined for streamlined analysis. A matrix coding structure was then developed to group data by ethnic background and theme, enabling the counting of occurrences and conversion into matrix format. This facilitated a co-occurrence analysis to identify how often themes appeared together within each ethnic group. The co-occurrence data were further transformed into a chord diagram format, ensuring completeness and consistency. Finally, a colour palette was applied, and themes were represented as labelled, colour-coded sectors, producing a chord diagram that clearly maps the interconnections and relationships among sociocultural practices (Figure 3).

The chord diagram revealed complex interconnections among sociocultural themes. Traditional rituals, medical practices, dietary habits, and support systems frequently co-occurred, indicating that certain practices are closely intertwined within maternal care. The analysis also highlighted ethnic diversity, showing unique patterns of theme co-occurrence across different ethnic groups. For example, Middle Eastern, African, and South Asian populations displayed distinct combinations of practices, emphasising the importance of culturally sensitive approaches in maternal healthcare. Some themes are more prominent and have stronger connections with multiple other themes. The strong presence of these themes, such as rituals and support systems, suggests that they are fundamental to the cultural framework of maternal care, shaping the core of maternal care across communities. The complexity of these interwoven practices shows the importance of culturally sensitive, holistic care to improve maternal health outcomes.

Network Analysis

Network analysis is a robust methodological and theoretical approach widely applied in healthcare research to explore complex structural and relational dynamics. In healthcare, network analysis has been instrumental in studying patient-physician interactions, care team collaborations, disease transmission, and health information dissemination [14,15].In qualitative thematic synthesis, network analysis is used to map and illustrate linkages between themes, codes, or individuals, showing deep interconnections that go beyond linear thematic descriptions. It uncovers hidden structures, supports data-driven intervention design, and improves the dissemination of complex relational data through intuitive, interpretable graphs [16]. Network maps are valuable tools in meta-synthesis for qualitatively synthesising complex relationships and patterns across multiple qualitative studies.

**Figure 4:**
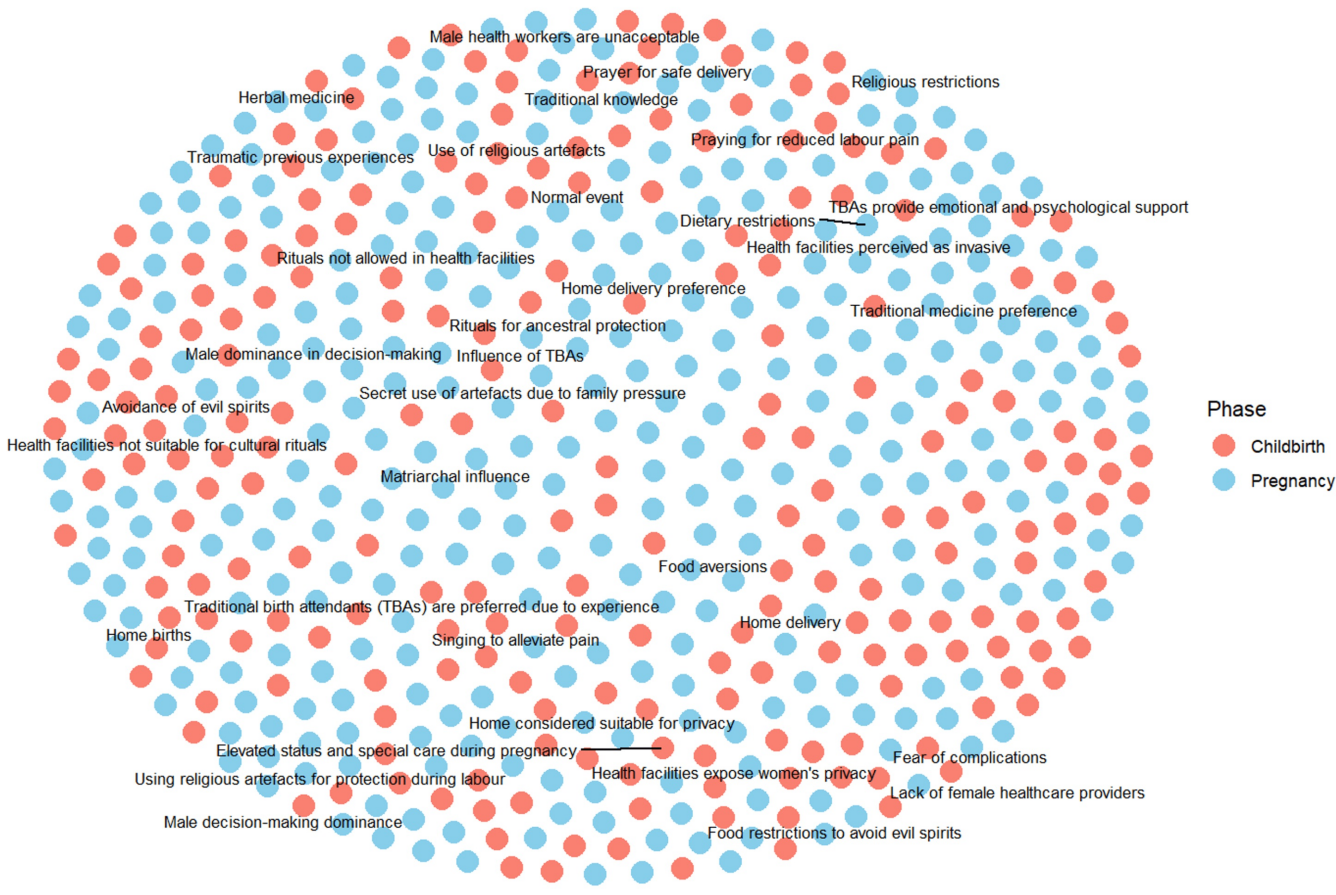
Network map depicting the sociocultural context of pregnancy and childbirth.

Network maps illustrate how themes, concepts, or sources are interconnected across different studies, allowing researchers to explore how ideas interrelate, converge, or diverge within the synthesised data. This helps illustrate the structure of qualitative evidence, highlights central or bridging themes, and reveals gaps or clusters in the research landscape. They also enhance transparency by showing how individual studies contribute to broader conceptual categories and support iterative interpretation during synthesis [1,17]. Data processing involved importing the dataset into R, selecting relevant columns, and combining themes into a single column for analysis. Themes were summarised by phase, and the 30 most frequent themes were identified. Node and link data frames were created to represent themes and their co-occurrences, which were then used to generate a network graph. The graph was visualised with nodes coloured by phase and labelled for clarity, allowing comparative analysis of relationships among themes (Figure 4).

The network analysis revealed interconnected sociocultural themes, with certain practices such as religious rituals, dietary habits, and traditional practices frequently co-occurring. Prominent themes varied by phase: childbirth-centred themes included emotional support from TBAs and prayer for reduced labour pain, while pregnancy-centred themes included dietary restrictions and male decision-making dominance. Phase-specific themes highlighted unique cultural concerns, such as fear of complications and unsuitability of health facilities for rituals during childbirth, and food aversions or elevated care during pregnancy. Overall, the network visualisation provides a broader depiction of the relationships and prominence of maternal health practices across cultural contexts.

Sentiment Analysis Using Geospatial Mapping

Sentiment analysis (SA) is the process of gathering and analysing people’s opinions, thoughts, and impressions regarding various topics, products, subjects, and services [18]. SA, also called opinion mining, is a part of natural language processing (NLP) that identifies whether a piece of text expresses a positive, negative, or neutral feeling. It started in the 1990s with studies on classifying viewpoints, adjective meanings, and subjective writing [19]. SA helps qualitative research and metasynthesis by systematically identifying emotions and opinions in large collections of health-related narratives. It complements traditional coding by uncovering hidden affective patterns, such as how patients and carers perceive treatments, services, or illness experiences.

SA integrated with Geographic Information System (GIS) mapping enhances meta-synthesis by combining NLP-driven sentiment extraction from qualitative data with geospatial analysis to contextualise emotions across the geographical landscape and their community’s variation [20]. This approach adds an effective layer to traditional spatial data, revealing hotspots and patterns of positive or negative sentiments linked to sociocultural and environmental factors [21]. By bridging qualitative themes with spatial visualisation, it supports mixed-methods synthesis, enriches contextual interpretation, and enables evidence-based decision-making through scalable, interactive maps that highlight place-based human experiences.

Key columns on study areas and sociocultural themes were selected from the dataset, and themes related to pregnancy and childbirth were combined into a single column for comparative analysis. Study areas were mapped to country names to maintain consistency, after which sentences from the themes were extracted and analysed to calculate sentiment values/scores. Mean sentiment scores were then summarised by country, providing an overall picture of sentiments linked to pregnancy and childbirth. GIS software (ArcGIS, QGIS) was used to map sentiment distributions, conduct hotspot analysis, and overlay thematic layers from meta-synthesis. Finally, these scores were merged with world map data, and an integrative visual was created using a colour gradient to display cross-country variations in sentiment.

**Figure 5:**
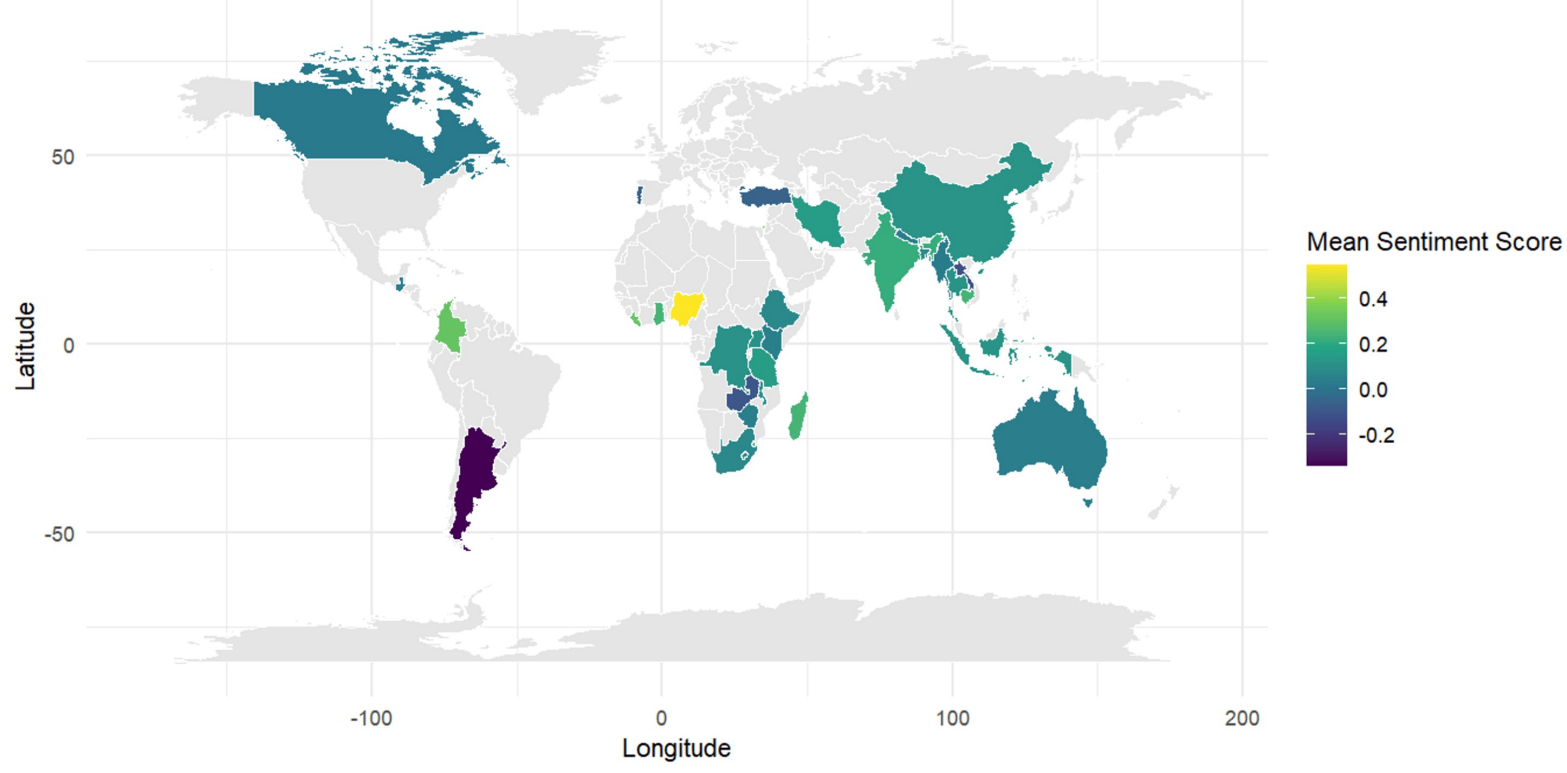
Geospatial sentiment analysis of cultural influence across global regions.

Based on the sentiment analysis map, clear variations emerge across regions in perceptions of pregnancy and childbirth practices. Nigeria and Ghana display the highest mean sentiment scores (around +0.35 to +0.4), shown in bright yellow, indicating strong positive sentiment and supportive practices. Meanwhile, European and some asian countries, such as Portugal and Turkey, fall within moderately positive ranges (+0.15 to +0.2), while countries such as Iran, Ethiopia, and Indonesia show near-neutral scores (around 0.0 to +0.1), suggesting a balance of positive and negative experiences pointing to diverse cultural influences. In contrast, Argentina and Colombia appear in shades of blue and purple with scores around -0.15 to -0.2, reflecting negative sentiments likely tied to cultural or systemic challenges (Figure 5). These spatial patterns highlight the diversity of perceptions globally and the importance of designing culturally sensitive and responsive, region-specific maternal health interventions that build on positive experiences while addressing negative ones.

These kinds of techniques offer a visual summary of intricate data sets to spot trends, correlations, and patterns. Even if the goal of visualisation is to make sense of the massive volume of data, it comes with its challenges. Misinterpretation, cognitive overload, inconsistent standards, and the requirement for expert, precise data understanding are still issues. The observer shouldn't be overwhelmed by charts or have important details obscured [22]. Future data visualisation exploration would be beneficial to enhance evidence-based research presentation and interpretation. While sentiment analysis offers scalable insights into affective patterns across large qualitative datasets, its application across culturally and linguistically diverse contexts has inherent limitations. Cultural nuance, contextual meaning, and translation variability may influence sentiment classification, potentially oversimplifying complex emotions or culturally embedded expressions. These findings should therefore be interpreted as indicative patterns rather than definitive measures of lived experience.

This study methodology does carry its limitations. The qualitative studies included were heterogeneous in design, context, and reporting quality, and variations in reporting quality may influence the comparability of themes. The synthesis relies on secondary data and published interpretations, which can restrict contextual depth and increase the risk of bias. Visualisation techniques, while enhancing interpretability, may inadvertently oversimplify sociocultural complexity or obscure intra-cultural variation. Geographic representation was also uneven across included studies, influencing sentiment distributions and limiting generalisability. Broadly, metasynthesis as a methodological approach is constrained by its dependence on authors’ interpretations, the potential loss of contextual richness during abstraction, and the challenges of merging diverse qualitative traditions into coherent higher-order themes. Despite these limitations, rigorous and thoughtful application of visualisation methods enriches qualitative evidence synthesis and supports a more nuanced understanding of a health determinant.

## Conclusions

Data visualisation functions as a critical analytical bridge in qualitative meta-synthesis by transforming complex narrative evidence into interpretable, relational visual forms. From early public health exemplars such as John Snow’s 1854 cholera map to contemporary visual analytics, including Sankey, chord, network, and sunburst diagrams, visualisation has demonstrated its capacity to reveal patterns, flows, and connections that may remain obscured in textual synthesis alone. This review demonstrates how strategically selected visualisation techniques can enhance the synthesis of sociocultural influences on pregnancy and childbirth by supporting cross-study comparison, depicting thematic interrelationships, and improving the accessibility of complex qualitative findings.

The effectiveness of visual analytics in qualitative synthesis, however, depends on clear analytical intent and methodological rigour. Visualisation is not a neutral representational tool; rather, its interpretive value lies in the alignment between research questions, synthesis logic, and the chosen visual form. When combined with transparent analytic procedures, visual analytics strengthens evidence integration and supports culturally sensitive interpretation relevant to clinical practice, health systems, and policy contexts. By making sociocultural patterns visible and interpretable, these techniques help identify gaps in understanding, inform context-specific interventions, and guide evidence-based decision-making in maternal health. Future work should continue refining visual analytics approaches to preserve contextual richness, enhance interpretive clarity, and maximise their applicability across diverse qualitative datasets, ensuring that meta-synthesis findings remain both methodologically robust and policy-relevant.
